# Commentary: The Dynamics of Aerotaxis in a Simple Eukaryotic Model

**DOI:** 10.3389/fcell.2022.844812

**Published:** 2022-03-02

**Authors:** Jean-Paul Rieu, Olivier Cochet-Escartin, Christophe Anjard, Mete Demircigil, Vincent Calvez

**Affiliations:** ^1^ Institut Lumière Matière, UMR5306, Université Lyon 1-CNRS, Université de Lyon, Villeurbanne, France; ^2^ Institut Camille Jordan, UMR5208, Université Lyon 1-CNRS, Université de Lyon, Villeurbanne, France

**Keywords:** Self-generated gradients, Aerotaxis, Collective migration, Oxygen sensing, Dictyostelium discoideum

**A Commentary on**

**The Dynamics of Aerotaxis in a Simple Eukaryotic Model**

*by Biondo, M., Panuzzo, C., Ali, S. M., Bozzaro, S., Osella, M., Bracco, E., and Pergolizzi, B. (2021). Front. Cell Dev. Biol. 9:720623. doi:* *10.3389/fcell.2021.720623*

We read with interest the article by Biondo et al. (2021) in Frontiers in Cell and Developmental Biology, “*The Dynamics of Aerotaxis in a Simple Eukaryotic Model*.” Reproducing the confinement assay we published in eLife earlier this year (Cochet-Escartin et al., 2021) with the same cell line, they found the same emergent behavior, i.e., the propagation of a ring of cells, which they named corona, from a dense, confined colony through the self-generation of oxygen gradients by cell consumption. The authors claimed that cell division plays no role in the phenomenon, whereas in our study, we insisted on its important role.

This message is wrong. In this commentary, we first clarify that ring formation is independent on cell division but that ring propagation over long times depends on it. Second, we discuss the possible experimental biases that may have led the authors to this conclusion.

**FIGURE 1:**
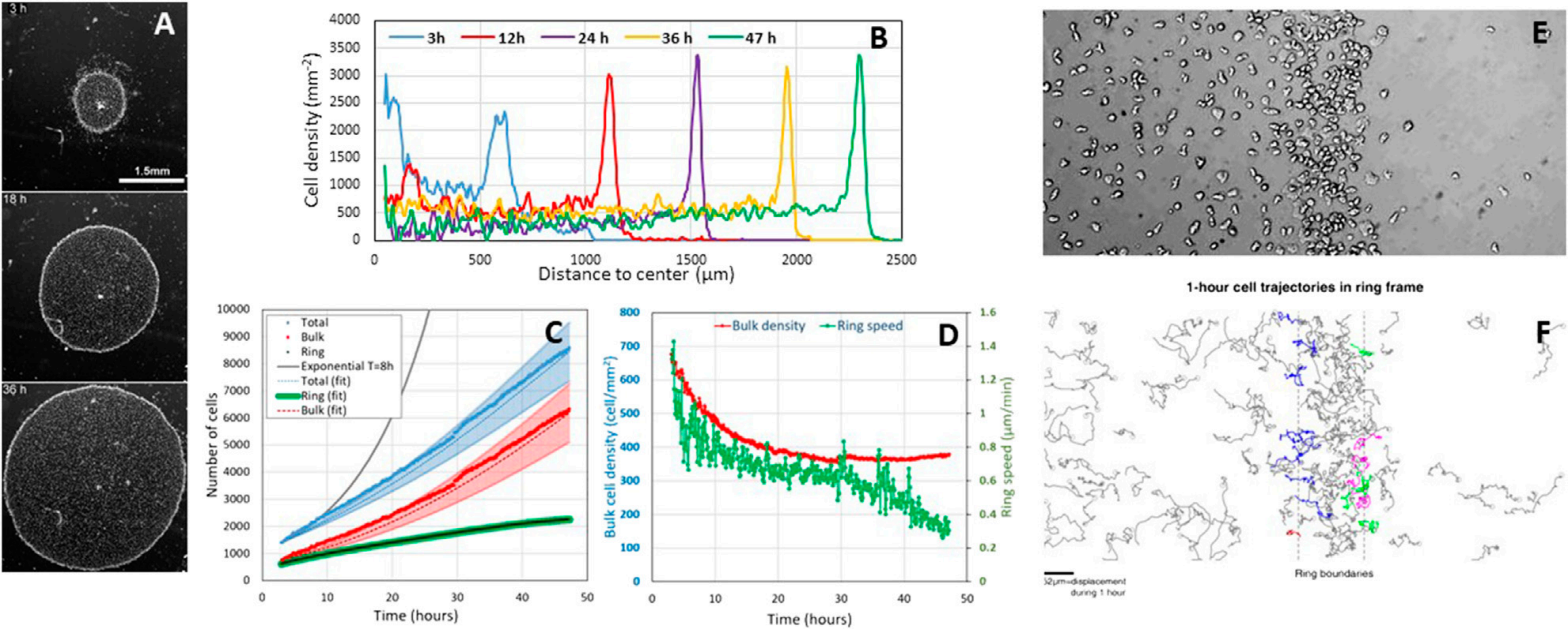
Cell proliferation during the aerotactic expansion of a small confined colony of vegetative *Dictyostelium* cells with an initial number of cell *N*(0 h) *=* 1,000 and an initial radius *R*(0 h) = 600 µm. **(A)** Snapshots at 3, 18, and 36 h showing the propagation of an external dense ring of cells mowing outwardly. Scale bar, 1.5 mm. **(B)** Corresponding stationary radial density profiles. **(C)** Measurements of the number of ring and bulk cells as well as the sum of the two subpopulations (total). The ring cells were estimated by measuring their density per unit length and multiplying by the perimeter. The bulk and total cell numbers have been fitted by Eqs 1, 2 (dotted lines) with $\varphi_{R\to B}=12\;\text{cells/mm/h}$ and error bars correspond to a 20% error on $\varphi_{R\to B}$ . **(D)** Ring speed and bulk cell density measurements over time. **(E,F)** Close view of the ring region to estimate the cell exchanges between the ring and the bulk. **(F)** Cell trajectories lasting 1 h in the ring frame. Ring borders are depicted by the dashed line. Blue, red, green, and purple trajectories correspond to trajectories that escape the ring toward the bulk, reach the ring from the bulk, reach the ring from the front, and escape the ring from the front, respectively. Overall, the net flux of cell from ring to front is zero; the net flux of cell from ring to bulk is approximately $\varphi_{R\to B}=12\;\text{cells/mm/h}$ .

**Cell division is not necessary for ring formation but is necessary for its sustained propagation**. Biondo et al. observed exactly the same collective phenotype as us for the confined colony in a starving buffer that prevents cell division. A ring forms internally, but as soon as it reaches the colony edge (at ∼6 h), it stops and cells aggregate (compare Movie 3 of Biondio et al. with our movie M6 and Figure 5; Supplementary Figure 2, Cochet-Escartin et al.). In contrast, in a nutrient medium, the ring propagates far away from the initial colony for days (see Figure 1A below). Biondo et al. neither commented on this fundamental difference between the two conditions nor on our model that demonstrates that cell division is necessary to maintain ring propagation even if it contributes little to the expansion speed (Figures 5A,B and Eq. 6, Cochet-Escartin et al.). Independently of any model, a simple mass balance equation for the total cell number N with NB cells in the bulk region (core) and NR cells in the ring region invalidates Biondo's assertion that division plays no role:

$$N\left(t\right)=N_{B}\left(t\right)+N_{R}\left(t\right)=\rho_{B}\pi {\left(R\left(t\right)-L\right)}^{2}+\rho_{R}2\pi R\left(t\right)L$$ (1)

Using the experimental observations (Figures 1D,E, Supplementary Figure S3B in Cochet-Escartin et al., Figures 1B–D below) that the ring width *L* and density *ρ*_*R*_ and bulk density *ρ*_*B*_ are constant, and that the ring radius R is expanding at constant speed *R(t) = R*_*0*_ *+ σt,* we predict that $N_{B}\left(t\right)$ increases faster with time (i.e., as $\;\;R^{2}∼t^{2}$ ) than $N_{R}\left(t\right)$ (i.e., as  LR∼t ). Experimentally, up to 30 h, $N_{B}\left(t\right)$ increases faster than linearly with time while *N*_*R*_ increases linearly (Figure 1C). Initially *N*_*B*_ */N*_*R*_ = 1, but after 24 h, *N*_*B*_ */N*_*R*_ = 1.8, and after 47 h, *N*_*B*_ */N*_*R*_ = 2.8 (Figure 1C). Hence, *N*_*B*_ largely contributes to the overall cell number increase *N(t)*. By comparison, Biondo et al. assume a constant $N_{R}\left(t\right)$ , and they do not consider bulk cells at all.

**Cell divisions hold in the ring**. Our confined colony grows slower than exponentially (see solid black line with a typical 8 h doubling time (d’Alessandro et al., 2016) in Figure 1C), but it grows (i.e., *N*(47 h)*/N*(0 h) = 8.5). In Cochet-Escartin et al., we propose a go-or-grow model where aerotaxis holds at low O_2_ and cell division at high O_2_. The threshold is around 1% O_2_ as estimated by direct aerotaxis investigations using microfluidic devices (Cochet-Escartin et al.) and from literature values for cell division in hypoxic conditions (Schiavo and Bisson, 1989; West et al., 2007). Such value corresponds to the O_2_ level measured in the ring (Cochet-Escartin et al.). Hence, divisions occur mostly in the ring, but ring cells are constantly transferred to the bulk to maintain a constant $\rho_{B}$ while *R* is increasing. This transfer occurs in our models (see Figure 7 of Cochet-Escartin et al.), but perhaps it was not sufficiently supported by data. In Figures 1E,F, we present manually tracked trajectories in the ring frame. A few ones displayed with a green or purple color enter or escape the outward ring position, canceling any ring-to-front flux. Far more trajectories are directed backward (i.e., ring to bulk, in blue). Interestingly, the measured flux of such a cell transfer, $\varphi_{R\to B}=12\;\text{cells/mm/h}$ , explains fairly well the bulk cell number increase using the following equation:

$$dN_{B}=2\pi R\phi_{R\to B}dt$$ (2)

The fit is displayed in Figure 1C.

**Biondo et al. may have caught a transient regime only**. Biondo et al. measured 3% and 5% O_2_ in the bulk and ring regions, respectively (Supplementary Figure S1). Above 2% O_2_, aerotaxis should not hold (Cochet-Escartin et al.); the division rate is fairly the same as in normoxic conditions (Schiavo and Bisson, 1989; West et al., 2007). A possible reason for this discrepancy is that O_2_ is overestimated. Their measurements were performed with a commercial sensing film that is not compatible with transmission microscopy, contrary to the technology we developed in Cochet-Escartin et al. They may have a different confinement on plastic (their usual experimental condition) than on the sensing film. A loose (resp. tight) confinement may generate a higher (resp. lower) O_2_ value under the colony. They also made colonies with a huge amount of cells (50,000 instead of 1,000 and 2,000 in our case). As the self-generated O_2_ field depends on the consumption of every cell, we expect a huge degree of hypoxia. Finally, they never reached a stationary expansion regime due to the large initial excess of inner cells. That excess density slowly decreases with time as visible on their kymograph. We have actually simulated a moderate bulk cell excess in our work (Figure 4 of Cochet-Escartin et al.) which is also transiently visible at 3 h in Figure 1B. Such an inner cell mass transfer has to be taken into account to establish a correct mass balance equation, and the only *L(t)R(t)* quantity tested by Biondo et al. is clearly not sufficient to draw a conclusion on cell divisions.
